# Genetic obesity increases pancreatic expression of mitochondrial proteins which regulate cholesterol efflux in BRIN-BD11 insulinoma cells

**DOI:** 10.1042/BSR20181155

**Published:** 2019-03-22

**Authors:** Anna-Maria Caridis, Richard J. Lightbody, Jamie M.R. Tarlton, Sharron Dolan, Annette Graham

**Affiliations:** Department of Biological and Biomedical Sciences, School of Health and Life Sciences, Glasgow Caledonian University, Glasgow, United Kingdom

**Keywords:** Cholesterol, high-density lipoprotein, Sterol 27-hydroxylase

## Abstract

Pancreatic β-cells are sensitive to fluctuations in cholesterol content, which can damage the insulin secretion pathway, contributing to the aetiology of type 2 diabetes mellitus. Cholesterol efflux to (apo)lipoproteins, via ATP-binding cassette (ABC) transporter A1 (ABCA1), can prevent intracellular cholesterol accumulation; in some peripheral cells, ABCA1-dependent efflux is enhanced by promotion of cholesterol trafficking to, and generation of Liver X receptor (LXR) ligands by, mitochondrial sterol 27-hydroxylase (Cyp27A1 (cytochrome P450 27 A1/sterol 27-hydroxylase)) and its redox partners, adrenodoxin (ADX) and ADX reductase (ADXR). Despite this, the roles of mitochondrial cholesterol trafficking (steroidogenic acute regulatory protein [StAR] and 18-kDa translocator protein [TSPO]) and metabolising proteins in insulin-secreting cells remain wholly uncharacterised. Here, we demonstrate an increase in pancreatic expression of Cyp27A1, ADXR, TSPO and LXRα, but not ADX or StAR, in obese (*fa/fa*) rodents compared with lean (*Fa/?*) controls. Overexpression of Cyp27A1 alone in BRIN-BD11 cells increased *INS2* expression, without affecting lipid metabolism; however, after exposure to low-density lipoprotein (LDL), cholesterol efflux to (apo)lipoprotein acceptors was enhanced in Cyp27A1-overexpressing cells. Co-transfection of Cyp27A1, ADX and ADXR, at a ratio approximating that in pancreatic tissue, stimulated cholesterol efflux to apolipoprotein A-I (apoA-I) in both basal and cholesterol-loaded cells; insulin release was stimulated equally by all acceptors in cholesterol-loaded cells. Thus, genetic obesity increases pancreatic expression of Cyp27A1, ADXR, TSPO and LXRα, while modulation of Cyp27A1 and its redox partners promotes cholesterol efflux from insulin-secreting cells to acceptor (apo)lipoproteins; this response may help guard against loss of insulin secretion caused by accumulation of excess intracellular cholesterol.

## Introduction

The cluster of metabolic abnormalities found associated with type 2 diabetes mellitus (T2DM), including insulin resistance (IR), central obesity, high blood pressure and hypertriglyceridaemia, are associated with low levels of high-density lipoprotein (HDL), and with dysfunctional HDL is unable to exert the multiple protective mechanisms ascribed to this lipoprotein (reviewed by Vollenweider et al. [1]). Evidence links HDL with improved insulin sensitivity and glucose utilisation in skeletal muscle [2], and beneficial effects of HDL on insulin secretion have been reported *in vivo* [2–4] and *in vitro* in some [5] but not all studies [6–8]. HDLs can protect against β-cell apoptosis, which can be triggered by an array of endoplasmic reticulum (ER) stressors [8–10]. Maintaining ER protein folding and trafficking is obviously critical in sustaining insulin secretion in the face of these challenges [9,10].

One key function of HDL is to modulate cholesterol homoeostasis: cholesterol levels within β-cells must remain within defined limits to maintain insulin secretion [11,12], while the accumulation of free cholesterol within cells triggers ER stress [13,14]. Apolipoprotein (apo) A-I (ApoA-I), the major apolipoprotein in HDL, interacts with ATP-binding cassette (ABC) transporter A1 (ABCA1) to initiate cholesterol efflux [15], while ABC transporter G1 (ABCG1) transfers cholesterol and phospholipids to HDL [16,17]. Knockout studies in mice indicate that ABCG1 aids the enrichment of insulin secretory granules with cholesterol needed for their formation and trafficking to the plasma membrane [18], while ABCA1-mediated cholesterol efflux is involved in their exocytosis [19–21]. The expression of both transporters is regulated by nuclear Liver X Receptors (LXR α/β), activated by endogenous oxysterol ligands that can be derived from the cholesterol biosynthetic pathway [22,23] or the oxidative metabolism of cholesterol by sterol 27-hydroxylase (CYP27A1) within mitochondria [24,25]. The latter pathway also provides an alternate route for elimination of excess cholesterol from cells in the periphery, via delivery of oxysterol to the liver for excretion [26].

The rate-limiting step governing the activity of CYP27A1 and its redox partners, adrenodoxin (ADX) and ADX reductase (ADXR) [27,28] is reported to be the delivery of cholesterol from the outer to the inner mitochondrial membrane [29,30], a process facilitated by steroidogenic acute regulatory protein (StAR; STARD1) [31–33] and 18-kDa translocator protein (TSPO) although the role of the latter remains controversial [34–38]. Overexpression and/or ligation of these proteins in macrophages can increase cholesterol efflux to (apo)lipoproteins, enhance ABCA1- and ABCG1-dependent cholesterol efflux and reduce neutral lipid mass and inflammation, via a mechanism that involves activation and/or induction of LXRα, and peroxisome proliferator activated receptor α (PPARα) [39–42].

Despite the presence of CYP27A1 in human pancreatic β-cells and islets [43], the function of mitochondrial cholesterol trafficking and metabolising proteins in insulin-secreting cells remain entirely uncharacterised. The aim of the present study was to examine the impact of obesity on pancreatic expression of these mitochondrial proteins (cytochrome P450 27 A1/sterol 27-hydroxylase (Cyp27A1), ADX, ADXR, StARD1, TSPO, LXRα) in the Zucker (*fa/fa*) rodent model of genetic obesity and their function in regulation of cholesterol efflux and insulin release in BRIN-BD11 insulinoma cells.

## Materials and methods

### Materials

ApoA-I and HDL were purchased from Athens Research and Technology (Georgia, U.S.A.). All other materials were from U.K. companies or U.K. suppliers for the companies indicated. Radiochemicals were purchased from PerkinElmer, Tri-Fast from PeqLab and cDNA synthesis kits from Bioline. Rabbit monoclonal antibodies to Cyp27A1, ADX and LXR, and polyclonal antibodies to TSPO, ADXR and ABCA1 were purchased from Abcam; rabbit polyclonal antibody to StarD1 was from Santa Cruz Biotechnology. Sodium pentobarbital was supplied by J.M. Loveridge PLC (Southampton), tissue culture reagents from Lonza and sterile tissue culture plastics from Greiner. The clones for Cyp27A1, ADX and ADXR (pCMV) were from Origene.com via Cambridge Biosciences. Complete™ protease inhibitor cocktail was purchased from Roche, and chemicals, TLC plates and solvents from Sigma–Aldrich.

### Experimental animals

Heterozygous Zucker rats (*Fa/fa*) were supplied by Harlan Laboratories (Bicester, U.K.) and a breeding colony established. Zucker rats were maintained in group (*n*=3) cages on sawdust bedding, on a 14-h light/10-h dark cycle with standard chow and water provided *ad libitum*. Male lean (*Fa/?*; *n*=9) and obese (*fa/fa*; *n*=5) rats were terminally anaesthetised at 4 months, using pentobarbital sodium (100 mg.kg^−1^, I.P.). Body weight was measured prior to commencement of study, then weekly thereafter. Blood was collected by cardiac puncture, and plasma levels of glucose and lipids determined, as described [44]; levels of serum insulin were determined using an ELISA supplied by Millipore. Principles of laboratory animal care (NIHA publication number 85-23, revised 1985 (http://grants1.nih.gov/grants/olaw/references/phspol.htm) were followed. The study was approved by the institution’s Animal Ethics and Welfare Committee, and procedures performed according to the U.K. Animals (Scientific Procedures) Act, 1986, and tissues harvested for multiple studies to reduce usage.

### Preparation of tissue samples

Samples (150–200 mg) of pancreas were suspended in Dulbecco’s PBS (1 ml), using a Beadbeater (Thistle Scientific, U.K.), and protein lysates were prepared in RIPA buffer plus Complete™ protease inhibitor cocktail.

### Cell culture and experiments

Rodent BRIN-BD11 insulinoma cells (ECACC 10033003) cells were maintained in RPMI-1640 medium containing foetal bovine serum (10% v/v) and penicillin/streptomycin (50 U/ml and 50 μg/ml respectively), at 37°C in a humidified atmosphere of 95% air and 5% CO_2_. All experiments were performed in RPMI 1640 medium containing 11 mM glucose, unless otherwise indicated. Transfection of BRIN-BD11 cells with 2 μg of empty vector (EV; pCMV.Neo) or the same vector encoding full-length rodent Cyp27A1, ADX or ADXR was achieved using Amaxa Nucleofector-II (Kit V, protocol G010). Stable populations were selected using G418 (400 μg.ml^−1^), and cellular viability assessed by conversion of MTT ((3-(4,5-dimethylthiazol-2-yl)-2,5-diphenyltetrazolium bromide) tetrazolium) to formazan [45]. Mitochondrial membrane potential was assessed using tetramethylrhodamine ethyl ester (TMRE), as previously described [45]. Efflux of cholesterol to apoA-I (10 μg.ml^−1^), HDL (10 μg.ml^−1^) and human serum (1%, v/v) was assessed in cells labelled with 0.5 μCi.ml^−1^ [^3^H]cholesterol, as described [41,42,46]. For measurement of lipid synthesis, cells were cultured in serum-free RPMI-1640 in the presence of [1-2 ^14^C]acetate (0.5 µCi/ml) [42,46].

### Lipid analyses

Cellular lipids were extracted using hexane:isopropanol (3:2, v/v), as detailed [41,42,46] and extracts dried under N_2_ before resuspension in isopropanol. Lipids labelled with [^14^C]acetate were separated by T.L.C. using petroleum ether: diethyl ether: glacial acetic acid (90:30:1 by vol.) as the mobile phase. Lipids were identified by comparison with authentic standards, and dpm determined by scintillation counting (Hidex 300SL). Cholesterol mass was measured by colorimetric assay, as previously described [41,42,46].

### Protein analyses

Protein lysates from pancreas and insulinoma cells were separated using 10% (w/v) SDS/PAGE gels, transferred to nitrocellulose membranes and probed using rabbit monoclonal or polyclonal antibodies to Cyp27A1 (1:1000), StarD1 (1:1000), TSPO (1:1000), ADX (1:1000), ADXR (1:500) and tubulin (1:1000) as described previously [41,42,46], except that fluorescently labelled secondary antibodies (LI-COR) were employed and bands were quantified using LI-COR Odyssey FC and Image Studio software. Mitochondria were isolated from insulinoma cell lines using a kit supplied by Abcam (ab110170).

### Statistical analysis

All values indicate mean ± S.E.M.; *n* denotes number of independent determinations. Significant (*P*<0.05) differences were determined using Student’s two-way *t* test when testing for significance between two groups of data, and one-way or repeated measures ANOVA and post-tests, when testing experiment with multiple outcomes, as previously [42]; repeated measures ANOVA was employed for paired experimental data.

## Results

### Pancreatic expression of mitochondrial cholesterol trafficking and metabolising proteins in obese (fa/fa) rats

Expression of mitochondrial proteins involved in the transport and metabolism of cholesterol were examined in pancreatic tissue isolated from 4-month old obese Zucker (*fa/fa*) male rats, compared with lean (*Fa/?*) male rats. At this age, *fa/fa* rats are normoglycaemic, but exhibit weight gain and hyperinsulinaemia, hyperlipidaemia and hepatic lipid accumulation [44] compared with lean controls. Obese (*fa/fa*) male rats weighed significantly more than lean controls of the same age and gender (*P*<0.05), but blood glucose levels did not differ significantly between the groups (Table 1). Serum cholesterol and triglyceride levels were increased by 1.65-fold (*P*<0.01) and 2.01-fold (*P*<0.001), respectively, in obese male rats, compared with the lean controls; however, insulin was elevated by 17-fold (*P*<0.001) in obese rats, indicating a compensatory phase of increased insulin output in order to sustain effective glucose control (Table 1).

**Table 1:** Weight, serum glucose, cholesterol, triglyceride and insulin levels in 4-month old male obese Zucker (fa/fa) and lean (Fa/?) rats

	Lean male rats	Obese male rats	Significance
**Weight (g)**	441.1 ± 10.7 (*n*=9)	550.4 ± 31.2 (*n*=5)	*P*=0.029
**Serum glucose (mM)**	8.01 ± 0.25 (*n*=9)	11.16 ± 1.98 (*n*=5)	*P*=0.190
**Serum cholesterol (mM)**	3.94 ± 0.08 (*n*=9)	6.53 ± 0.31 (*n*=5)	*P*=0.0013
**Serum triglyceride (mM)**	1.56 ± 0.08 (*n*=9)	3.14 ± 0.19 (*n*=5)	*P*=0.0006
**Serum insulin (ng/ml)**	6.05 ± 0.71 (*n*=9)	103.2 ± 11.29 (*n*=5)	*P*=0.0010

Significance values derived from a two-tailed unpaired Student’s *t*test, Welch corrected.

**Figure 1:**
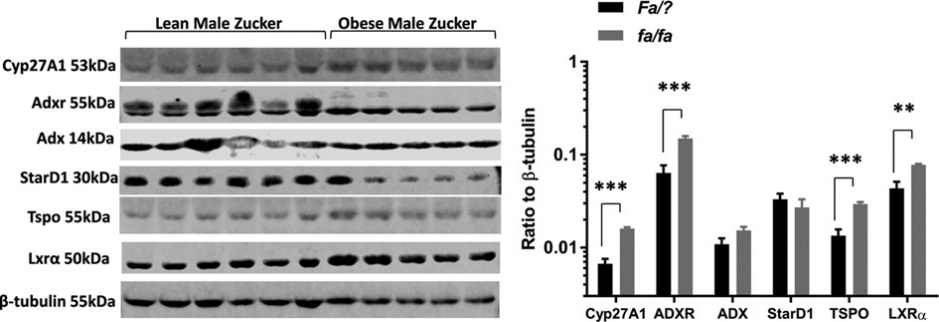
Pancreatic levels of Cyp27A1, ADXR, ADX, StAR, TSPO and LXRα relative to β-tubulin, in total pancreatic tissue extracted from lean (*Fa/?; n*=6) and obese (*fa/fa*; *n*=5) male rats measured by Western blotting Values are mean ± S.E.M.; **P<0.01, ***P<0.001 compared with lean controls.

Protein levels of Cyp27A1 and its redox partners ADX and ADXR in pancreatic tissue derived from lean and obese Zucker rats are shown in Figure 1. Levels of Cyp27A1 protein increased by 2.4-fold (*P*=0.0002) in pancreatic tissue derived from obese rodents, compared with lean controls. Pancreatic levels of ADXR protein also increased by 2.4-fold (*P*=0.0006) in tissue derived from obese rodents, compared with lean controls. The ADXR antibody also detected a second, higher molecular weight band in tissue derived from lean animals, but not from obese rodents, which remains uncharacterised, and was not included in the analyses performed here. Levels of ADX did not increase significantly between these groups (*P*=0.1255). The ratio of Cyp27A1/ADXR/ADX in pancreatic tissue therefore changes from 1:9.3:1.6 in lean animals to 1:9.4:0.95 in obese rats. Pancreatic levels of StAR and TSPO are also shown in Figure 1. Expression of StAR protein did not change significantly in obese animals, compared with lean controls, but levels of TSPO increased by 2.2-fold (*P*=0.0004) in the pancreas of obese, compared with lean, rodents. Finally, levels of LXRα protein increased by 1.8-fold (*P*=0.0067) in pancreatic tissue from obese animals, compared with lean controls (Figure 1), suggesting increased activation of LXR [58] is associated with enhanced Cyp27A1 expression.

Thus, altered pancreatic levels of Cyp27A1, ADXR, TSPO and LXRα are triggered in response to genetic obesity in rodents, a condition characterised by dyslipidaemia and hyperinsulinaemia (Table 1). These putative relationships were explored further in rodent BRIN-BD11 insulinoma cells; while human pancreatic islets and β-cells are known to express CYP27A1, rodent insulinoma cells do not, indicating that this protein is not an *essential* requirement for glucose-stimulated insulin secretion [43]. However, insulin release in BRIN-BD11 cells is responsive to modulation of cholesterol content [47] providing a suitable cellular context in which to examine the relationship between cholesterol trafficking and metabolising proteins and the cholesterol efflux pathway.

**Figure 2:**
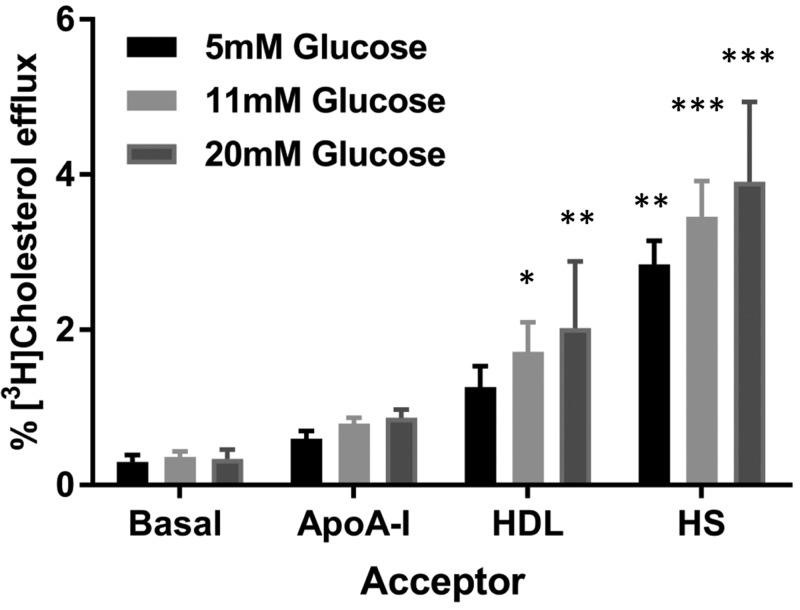
Efflux (2 h) of [^3^H]cholesterol to media (basal), apoA-I (10 μg.ml^−1^), HDL (10 μg.ml^−1^) and human serum (1%, v/v) from wild-type BRIN-BD11 insulinoma cells, in the presence of RPMI-1640 medium containing 5 mM (*n*=3), 11 mM (*n*=6) or 20 mM (*n*=3) glucose Values are mean ± S.E.M.; *P<0.05, **P<0.01 and ***P<0.001 compared with the basal control under the equivalent glucose concentration.

Cholesterol efflux (2 h) from wild-type BRIN-BD11 cells to acceptor (apo)lipoproteins over a range of glucose concentrations is shown in Figure 2; human serum contains not only acceptor (apo)lipoproteins, but also the enzymes and transfer proteins involved in the reverse cholesterol transport pathway [48]. In the presence of 5 mM glucose, efflux of [^3^H]cholesterol to human serum (1%, v/v) was significantly higher (9.5-fold; *P*<0.01) than that to basal media, but efflux to apoA-I (10 μg.ml^−1^) and HDL (10 μg.ml^−1^) was not significantly increased, suggesting glucose availability was limiting the efflux. However, in the presence of 11 mM glucose, efflux was stimulated by both HDL (4.8-fold; *P*<0.05) and human serum (9.6-fold; *P*<0.001), and the extent of these responses were not significantly altered by exposure to 20 mM glucose.

**Figure 3:**
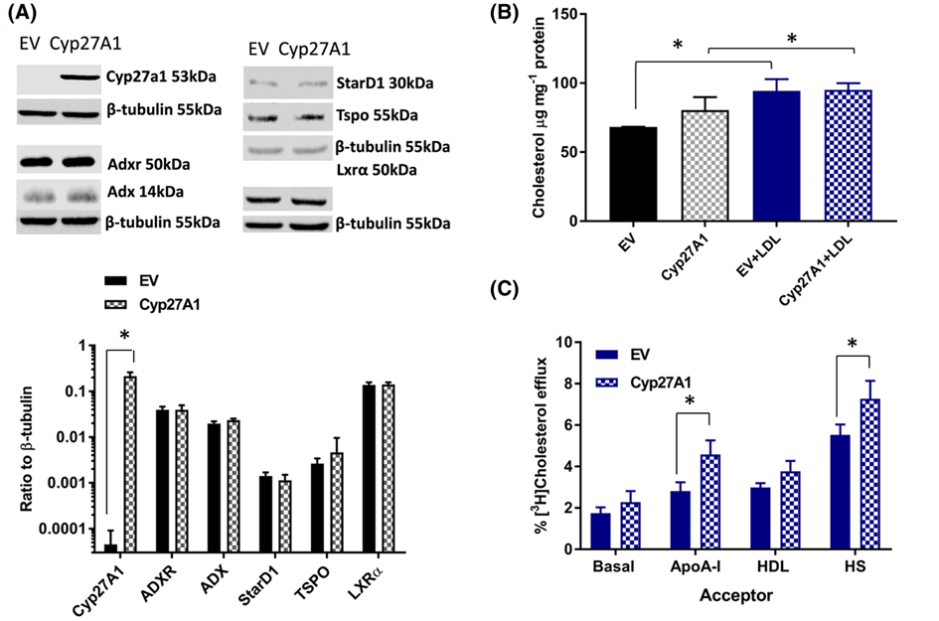
The impact of Cyp27A1 overexpression on cholesterol mass and efflux in BRIN-BD11 cells Representative Western blot images and levels of Cyp27A1, ADXR, ADX, StAR, TSPO and LXRα, relative to β-tubulin, in stable populations of BRIN-BD11 cells transfected with Cyp27A1, or the EV control are shown in (**A**). Values are mean ± S.E.M. of between three and five independent experiments; *P<0.05 compared with the EV control. The total cholesterol mass in EV and Cyp27A1 expressing cell populations under basal conditions, and following exposure to native LDL (100 μg.ml^−1^; 24 h) is shown in (**B**). Values are mean ± S.E.M., *n*=4; *P<0.05 for the comparisons indicated. Efflux of [^3^H]cholesterol from Cyp27A1 and EV cell populations, after exposure to native LDL (100 μg.ml^−1^; 24 h), to basal media, apoA-I (10 μg.ml^−1^), HDL (10 μg.ml^−1^) and human serum (1%, v/v), after 2 and 24 h, are shown in (**C**). Values are mean ± S.E.M., *n*=4; *P<0.05 for the comparisons indicated. Abbreviation: LDL, low-density lipoprotein.

Transient introduction (48 h) of full-length rodent Cyp27A1 (110-fold; *P*=0.002; *n*=6) into BRIN-BD11 cells significantly increased basal efflux (2 h) of [^3^H]cholesterol (EV; pCMV.6) 1.95 ± 0.24% compared with pCMV.6_Cyp27A1 2.60 ± 0.33%; *n*=6, *P*=0.010 in a paired two-tailed *t*test). Stable cell populations were generated, using the same pCMV.6 vectors (Figure 3A), to achieve a substantial (three orders of magnitude) increase in Cyp27A1 expression in BRIN-BD11 cells compared with the EV control. These cell lines exhibited very similar linear growth parameters, as judged by the cumulative rate of conversion of MTT into formazan over a period of 0–96 h in three independent experiments: EV 0.55 ± 0.04 μmol formazan/h (R^2^ = 0.99) compared with Cyp27A1 0.59 ± 0.15 μmol formazan/h (R^2^ = 0.88), with no significant difference noted (*P*=0.81) between these cell populations.

Protein levels of ADX, ADXR, StarD1, TSPO and LXRα in Cyp27A1 overexpressing cells are also shown in Figure 3A. In brief, no changes in the levels of these proteins were noted in Cyp27A1 expressing cells, compared with EV controls; the ratio of Cyp27A1/ADXR/ADX in the former was 1:0.184:0.109, indicating a marked excess of Cyp27A1 protein compared with its redox partners.

Biosynthesis of free cholesterol from [^14^C]acetate (24 h) did not change significantly (EV 1659.5 ± 336.6 pmol/mg cell protein compared with Cyp27A1 1405.1 ± 359.7 pmol/mg cell protein; *n*=6; *P*=0.071 in a two-tailed *t*test) in Cyp27A1 overexpressing cells; no changes in incorporation of [^14^C]acetate into the cholesteryl ester pool were noted (EV 490.3 pmol/mg cell protein compared with Cyp27A1 490.8 pmol/mg cell protein; *n*=6). Efflux of [^3^H]cholesterol to basal media, apoA-I, HDL or human serum in Cyp27A1 overexpressing cells at 2 h, and at 24 h, compared with the EV control is shown in Table 2; all acceptors significantly enhanced [^3^H]cholesterol efflux above the basal condition after 24 h in culture in both cell populations, but no significant differences between EV and Cyp27A1 overexpressing cells were noted under any of the conditions tested.

**Table 2:** The effect of overexpression of Cyp27A1 on [^3^H]cholesterol efflux to (apo)lipoprotein acceptors

Acceptor	[^3^H]Cholesterol efflux (2 h) EV	[^3^H]Cholesterol efflux (2 h) Cyp27A1	[^3^H]Cholesterol efflux (24 h) EV	[^3^H]Cholesterol efflux (24 h) Cyp27A1
**Basal**	0.64 ± 0.12% (*n*=3)	0.96 ± 0.32% (*n*=3)	6.44 ± 0.23% (*n*=15)	6.54 ± 0.31% (*n*=15)
**ApoA-I (10 μg.ml^−1^)**	1.76 ± 0.32% (*n*=3)	1.91 ± 0.34% (*n*=3)	8.76 ± 0.40%^2^ (*n*=15)	8.88 ± 0.45%^2^ (*n*=15)
**HDL (10 μg.ml^−1^)**	2.39 ± 0.24%^1^ (*n*=3)	2.47 ± 0.28%^1^ (*n*=3)	14.62 ± 1.10%^2^ (*n*=11)	14.59 ± 0.96%^2^ (*n*=11)
**Human serum (1%, v/v)**	3.84 ± 0.36%^2^ (*n*=3)	4.07 ± 0.61%^2^ (*n*=3)	23.34 ± 0.57%^2^ (*n*=11)	23.74 ± 0.72%^2^ (*n*=11)
**0.3 mM dbcAMP**	-	-	7.09 ± 0.55% (*n*=3)	5.75 ± 0.28% (*n*=3)
**0.3 mM dbcAMP+ ApoA-I (10 μg.ml^−1^)**	-	-	9.69 ± 0.77% (*n*=3)	8.21 ± 0.13% (*n*=3)
** 0.3 mM dbcAMP+HDL (10 μg.ml^−1^)**	-	-	16.57 ± 0.52%^3^ (*n*=3)	14.23 ± 1.01% (*n*=3)
**0.3 mM dbcAMP + human serum (1%, v/v)**	-	-	24.13 ± 0.31% (*n*=3)	23.75 ± 0.87% (*n*=3)
**10 μM FGIN-1-27**	-	-	7.53 ± 0.89% (*n*=3)	6.81 ± 0.37% (*n*=3)
**10 μM FGIN-1-27 + ApoA-I (10 μg.ml^−1^)**	-	-	8.13 ± 0.51% (*n*=3)	7.58 ± 0.38% (*n*=3)
**10 μM FGIN-1-27 + HDL (10 μg.ml^−1^)**	-	-	15.02 ± 1.03% (*n*=3)	14.85 ± 1.36% (*n*=3)
**10 μM FGIN-1-27 + human serum (1%, v/v)**	-	-	23.85 ± 0.60% (*n*=3)	23.97 ± 0.89% (*n*=3)

Values are the mean ± S.E.M. for the number of independent experiments indicated by ‘*n*’; each independent experiment was performed using triplicate wells of cultured cells. Significant differences in efflux due to each acceptor from basal (EV) and basal (Cyp27A1) controls are indicated. ^1^*P*<0.05. ^2^*P*<0.001. No differences in efflux were noted between EV and Cyp27A1 stable cell populations under any of the conditions tested. The significant difference in efflux to HDL, due to the presence of dibutyryl cAMP (0.3 mM; 24 h) in the EV stable population of cells, is indicated as ^3^*P*<0.05. No significant differences in efflux were found due to treatment with FGIN-1-27 (10 μM; 24 h) in paired experiments from EV or Cyp27A1 stable populations of cells.

The EV- and Cyp27A1-stable populations of BRIN-BD11 cells were treated with dibutyryl cAMP (0.3 mM), to stimulate the expression and activity of StAR [31,41], or with the TSPO ligand FGIN-1-27 (10 μM) [36,42], factors which increase mitochondrial cholesterol trafficking, LXRα activation and/or cholesterol efflux in other cell types. Cholesterol efflux (24 h) to HDL (10 μg.ml^−1^) was significantly increased in EV, but not Cyp27A1 cells, following preincubation with cAMP analogue (Table 2). Treatment with FGIN-1-27 (10 μM) did not significantly enhance cholesterol efflux (24 h) to apoA-I, HDL or HS in either cell line (Table 2); further, no consistent changes in efflux of cholesterol to acceptors were noted following transient introduction (48 h) of TSPO into Cyp27A1 and EV cell lines (*data not shown*).

By contrast, increasing the cholesterol content of EV and Cyp27A1 cell lines by preincubation with low-density lipoprotein (LDL) (100 μg.ml^−1^; 24 h) (Figure 3B) revealed an enhanced capacity for efflux (2 h) to apoA-I (1.63-fold; *P*<0.01) in Cyp27A1 overexpressing cells compared with EV controls. Efflux of cholesterol (2 h) to human serum also increased (1.32-fold; *P*<0.05) in Cyp27A1 cells compared with EV, but removal of cholesterol by HDL remained unchanged (Figure 3C). After 24 h, efflux of [^3^H]cholesterol to HDL and human serum were significantly higher than basal efflux in both cell populations, but only basal efflux was stimulated (EV 4.35 ± 0.53% compared with Cyp27A1 6.02 ± 0.68%; *P*<0.01; *n*=4) by Cyp27A1 expression compared with EV controls.

**Figure 4:**
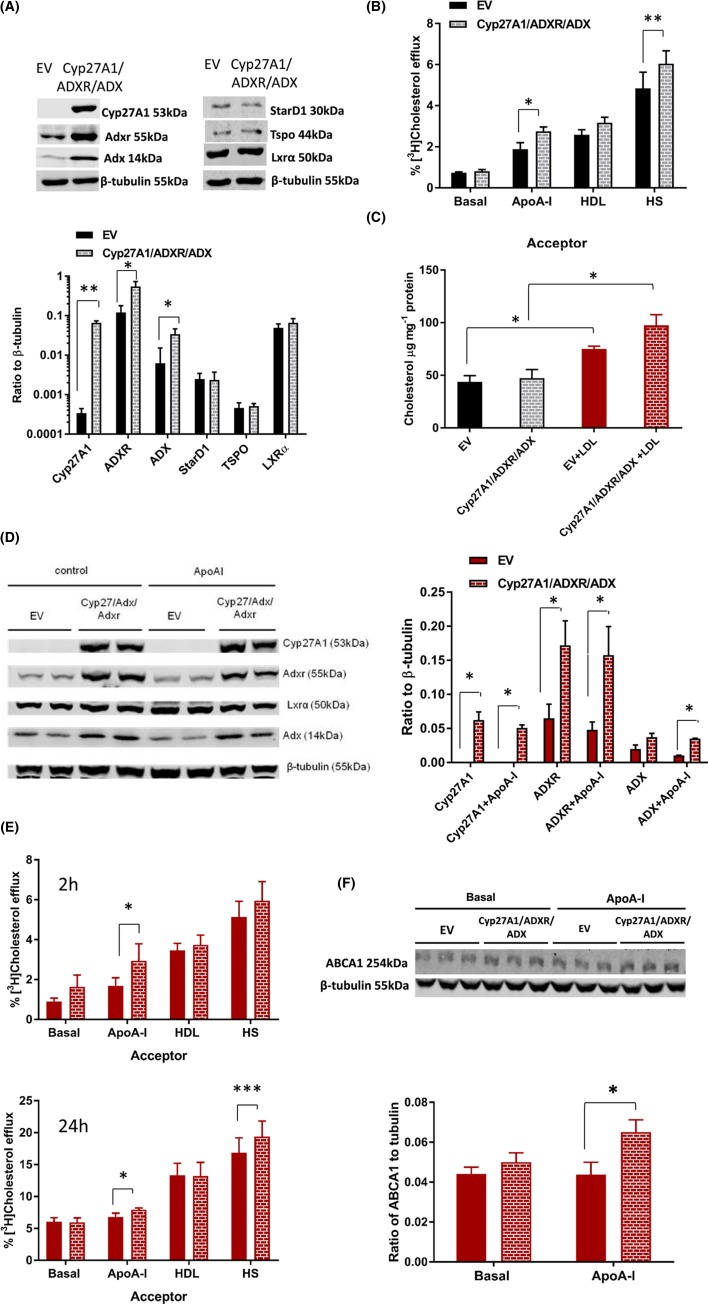
The impact of overexpression of Cyp27A1/ADX/ADXR on cholesterol mass and efflux in BRIN-BD11 cells Representative Western blot images and levels of Cyp27A1, ADXR, ADX, StAR, TSPO and LXRα, relative to β-tubulin, in stable populations of BRIN-BD11 cells transfected with Cyp27A1/ADX/ADXR, or the EV control are shown in (**A**). Values are mean ± S.E.M. of seven independent experiments; *P<0.05, **P<0.01 compared with the EV control. Efflux of [^3^H]cholesterol to basal media, apoA-I (10 μg.ml^−1^), HDL (10 μg.ml^−1^) and human serum (1%, v/v), after 2 h is shown in (**B**) (mean ± S.E.M.; *n*=4); *P<0.05 and **P<0.0.01 for the comparisons indicated. The total cholesterol mass in EV and Cyp27A1/ADXR/ADX expressing stable cell populations under basal conditions, and following exposure to native LDL (100 μg.ml^−1^; 24 h) is shown in (**C**). Values are mean ± S.E.M., *n*=3; *P<0.05 for the comparisons indicated. Representative Western blot images and protein levels of Cyp27A1, ADXR and ADX in these stable cell populations, after exposure to native LDL (100 μg.ml^−1^; 24 h), and in the presence and absence of apoA-I (10 μg.ml^−1^; 2 h) are shown in (**D**). Values are mean ± S.E.M., *n*=3; *P<0.05 compared with the EV control. Efflux of [^3^H]cholesterol from Cyp27A1/ADXR/ADX and EV cell populations, after exposure to native LDL (100 μg.ml^−1^; 24 h), to basal media, apoA-I (10 μg.ml^−1^), HDL (10 μg.ml^−1^) and human serum (1%, v/v), after 2 h (*n*=6) and 24 h (*n*=5), are shown in (**E**). Values are mean ± S.E.M., *n*=3; *P<0.05, ***P<0.001 for the comparisons indicated. Levels of ABCA1 protein, in EV and Cyp27A1/ADXR/ADX cell lines, after exposure to native LDL (100 μg.ml^−1^), in the presence and absence of apoA-I (10 μg.ml^−1^; 2 h) are shown in (**F**). Values are mean ± S.E.M., *n*=4; *P<0.05 for the comparison indicated.

In the above experiments, expression of Cyp27A1 was markedly increased without changes in expression of its redox partners. Stable cell populations were therefore generated using the same total amount of DNA (2 μg) and pCMV.6 clones expressing full-length rodent Cyp27A1, ADXR and ADX in a 1:1:1 ratio (Figure 4A), achieving a final protein ratio of Cyp27A1/ADXR/ADX of 1:8.36:0.52, which approximates that seen in pancreatic tissue from (*fa/fa*) Zucker rats (Figure 1). Again, these cell lines exhibited very similar linear growth parameters, as judged by the rate of conversion of MTT into formazan over a period of 0–96 h in three independent experiments: EV 0.75 ± 0.07 μmol formazan/h (R^2^ = 0.98) compared with Cyp27A1 0.65 ± 0.06 μmol formazan/h (R^2^ = 0.98), with no significant difference noted (*P*=0.34) between these cell populations. Altered mitochondrial cholesterol content was not observed in these cell lines (EV 0.225 ± 0.063 mg cholesterol/mg protein compared with Cyp27A1/ADXR/ADX 0.23 ± 0.055 mg cholesterol/mg protein; mean ± S.E.M.; *n*=4), and no changes in mitochondrial membrane potential were evident (EV 23231 ± 674 arbitrary fluorescent units (AFU) compared with Cyp27A1/ADXR/ADX 26056 ± 2420 AFU; mean ± S.E.M.; *n*=3).

Protein levels of StarD1, TSPO and LXRα in Cyp27A1/ADXR/ADX overexpressing cells are shown in Figure 4A. While significant increases in Cyp27A1 (*P*=0.015), ADXR (*P*=0.019) and ADX (*P*=0.002) were noted, no alterations in StarD1, TSPO or LXRα were evident. Despite the levels of Cyp27A1, relative to β-tubulin, being only a third of that in Figure 3A, cholesterol efflux (2 h) to apoA-I and human serum were stimulated in Cyp27A1/ADXR/ADX cells by 1.46-fold (*P*<0.05) and 1.25-fold (*P*<0.05) compared with EV control (Figure 4B). At 24 h, efflux to HDL and human serum were significantly elevated (*P*<0.001) above the basal condition in both cell lines, but no significant differences in cholesterol efflux were noted between these cell populations in three independent experiments (*data not shown*).

The EV and Cyp27A1/ADXR/ADX stable cell lines were then exposed to cholesterol (LDL; 100 μg.ml^−1^; 24 h), achieving enhanced cholesterol mass (Figure 4C). The levels of Cyp27A1, ADXR and ADX were measured in cholesterol-loaded cells, and in the presence and absence of apoA-I (10 μg.ml^−1^; 2 h). The Cyp27A1/ADXR/ADX ratio in cholesterol-loaded cells changed from that seen in Figure 4A, to 1:2.75:0.59 and 1:1.31:1.45 in the absence and presence of ApoA-I, respectively, primarily due to a decrease in the levels of ADXR (Figure 4D). Cholesterol efflux (2 and 24 h) under cholesterol-loaded conditions from EV and Cyp27A1/ADXR/ADX cell lines is shown in Figure 4E. Efflux to apoA-I (2 h) was enhanced by 1.74-fold (*P*<0.05) in the Cyp27A1/ADXR/ADX cell population, compared with the EV control, while modest increases in efflux to apoA-I and humans were also noted in the same cell populations after 24 h. Protein levels of ABCA1 were measured in these cell lines under basal and cholesterol-loaded conditions (Figure 4F), in the presence and absence of apoA-I (10 μg.ml^−1^; 2 h). No significant differences in expression of ABCA1 were noted in cells which were not exposed to a cholesterol load (*data not shown*); however, in the presence of apoA-I, ABCA1 levels were significantly increased (1.5-fold; *P*<0.05) in cholesterol-loaded Cyp27A1/ADXR/ADX cells, compared with the equivalent EV controls.

**Figure 5:**
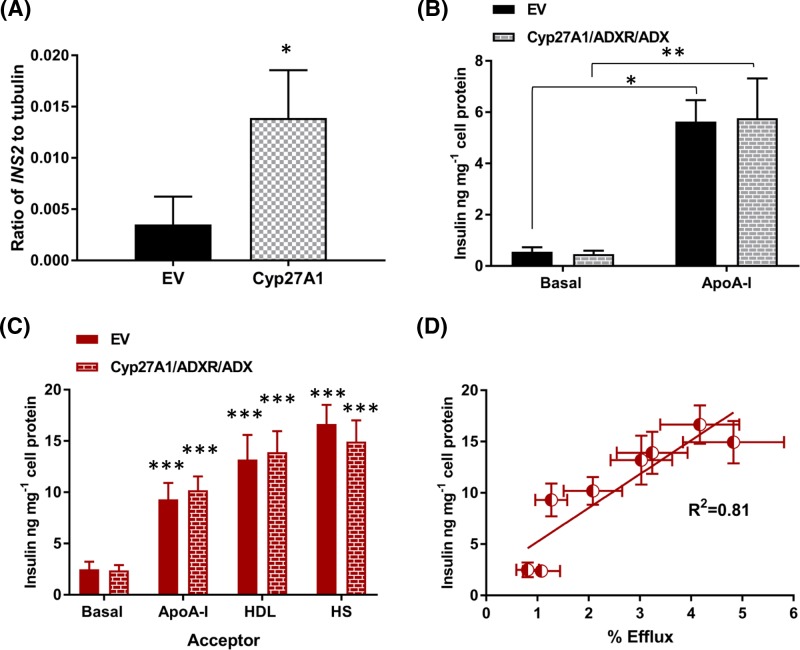
The impact of overexpression of Cyp27A1 and/or ADX/ADXR on insulin gene expression and release from BRIN-BD11 cells The expression of the *INS2* gene, relative to β-tubulin, in EV and Cyp27A1 expressing stable populations is shown in (**A**); values are mean ± S.E.M., *n*=6; *P<0.05 compared with the EV control. Release of insulin (30 min) into the media above EV and Cyp27A1/ADXR/ADX cells, in response to apoA-I (10 µg.ml^−1^) is shown in (**B**); values are mean ± S.E.M., *n*=3; *P<0.05, **P<0.01 for the comparisons indicated. Release of insulin under efflux conditions (2 h) into basal media, or media supplemented with apoA-I (10 μg.ml^−1^), HDL (10 μg.ml^−1^) and human serum (1%, v/v) following incubation in the presence of native LDL (100 μg.ml^−1^, 24 h), is shown in (**C**), with the correlation between insulin release and % efflux shown in (**D**). Values are mean ± S.E.M., *n*=5; ***P<0.001 compared with the relevant basal control.

Overexpression of Cyp27A1 alone was sufficient to increase expression of the insulin gene (*INS2*) by nearly four-fold (*P*<0.05) (Figure 5A); however, the release of insulin (30 min) into media above EV and Cyp27A1/ADXR/ADX cell populations was stimulated by the presence of apoA-I (10 μg.ml^−1^) to an equivalent extent (Figure 5B). No increase in gene expression of other secretory proteins (insulin-like growth factor (IGF), vascular endothelial growth factor (VEGF)) were noted in these cell lines (*data not shown*). When the same cell lines were preincubated with LDL (100 μg.ml^−1^; 24 h), the release of insulin under efflux conditions (2 h) was stimulated by the presence of apoA-I (*P*<0.001), HDL (*P*<0.001) and human serum (*P*<0.001), compared with the basal condition (Figure 5C); no insulin was detected in medium from cell-free controls containing the same concentrations of each cholesterol acceptor. No differences in the extent of insulin release stimulated by each acceptor were noted between each cell line but a significant (R^2^ = 0.81; *P*=0.002) relationship emerged between insulin levels in the medium and cholesterol efflux (Figure 5D). Finally, treatment with 0.8 mM octanol (2 h) was used to mobilise the plasma membrane cholesterol pool [49,50] in both stable cell populations, and insulin release measured in the presence of apoA-I. Compared with the vehicle control (ethanol, 0.1%, v/v), treatment with octanol significantly decreased insulin release in EV cells (19.72 ± 3.72 ng insulin.mg^−1^ cell protein compared with octanol 11.23 ± 3.58 ng insulin.mg^−1^ cell protein; *n*=3; *P*<0.01), but not in the Cyp27A1/ADXR/ADX cell population (16.4 ng insulin.mg^−1^ cell protein compared with octanol 11.55 ± 2.94 ng insulin.mg^−1^ cell protein; *n*=3).

## Discussion

The present study demonstrates that hyperinsulinaemia and hyperlipidaemia in normoglycaemic genetically obese male rodents is accompanied by increased protein expression of Cyp27A1, ADXR and TSPO, and nuclear transcription factor LXRα in pancreatic tissue. The relationships between changes in expression of these mitochondrial cholesterol trafficking and metabolising proteins, and the cholesterol efflux pathway, were then probed in rodent BRIN-BD11 insulinoma cells. Reconstitution of Cyp27A1 in BRIN-BD11 cells, which do not express this enzyme (unlike human β-cells) [43], increased the expression of *INS2*, without affecting lipid metabolism, in the absence of a cholesterol ‘load’. However, after exposure to LDL, enhanced cholesterol efflux to apoA-I and human serum were noted in Cyp27A1 expressing cells, compared with controls. Agents that bind and/or activate StAR and TSPO did not markedly impact cholesterol efflux, either in the presence or absence of Cyp27A1. Co-transfection of Cyp27A1, ADX and ADXR, in a ratio approximating that in obese pancreatic tissue, stimulated ABCA1-dependent cholesterol efflux to apoA-I in both basal and cholesterol-loaded cells, compared with controls. Insulin release into the media was stimulated by all acceptors to a similar extent, but proved more susceptible to mobilisation of plasma membrane cholesterol in control cells than in cells expressing Cyp27A1 and its redox partners.

Obesity has previously been linked to altered expression of Cyp27A1 in mice, in a tissue-specific manner: in male C57BL/6 mice fed a high-fat diet, hepatic expression of Cyp27A1 were found to be lower than in control mice, while the reverse was true in visceral adipose tissue, and has been associated with changes in vitamin D metabolism [51]. While pancreatic expression of Cyp27A1 has not previously been investigated, 27-hydroxycholesterol is a major product of cholesterol metabolism in adipocytes, and inhibition of Cyp27A1 activity or knockdown/deletion of *Cyp27A1* induces adipocyte differentiation [52,53]. It is possible that induction of Cyp27A1 may therefore be an autocrine or paracrine response to limit adipocyte hyperplasia during overfeeding, and could exert a similarly protective function in pancreatic tissue to prevent cholesterol accumulation in genetic obesity [52,53]. Loss of Cyp27A1 activity has also been linked to increased concentrations of active glucocorticoids [54], so one hypothesis could be that pancreatic induction of Cyp27A1 expression, possibly mediated by activation of glucocorticoid receptors [55,56], could help to reduce the impact of increased circulating concentrations of these stress hormones in obesity [57]. Alternatively, pancreatic induction of Cyp27A1 may contribute to the compensatory response leading to hyperinsulinaemia in *fa/fa* Zucker rats. We hypothesise that increased local (auto/paracrine) production of endogenous oxysterol ligands for LXRs [58], which diffuse within tissues and the bloodstream, may explain increased pancreatic expression of LXRE-dependent LXRs observed in obese rodents.

Regulation of ADX and ADXR expression has not previously been studied in pancreatic tissue, or the context of genetic obesity. However, the availability of ADXR and ADX can limit the activity of CYP11A1 [59,60], while co-transfection of both redox partners achieves the reverse [32,61] so that the altered ratio of Cyp27A1/ADXR/ADX in pancreatic tissue from obese rats may alter the efficiency of this cholesterol metabolising complex. By contrast, TSPO is repressed by obesity in white and brown adipose tissue [62], but up-regulated in the brain following administration of a high-fat obesogenic diet [63], and TSPO ligands have been linked with improved metabolic and/or inflammatory status in a number of chronic disease states [52,53,64]. The increase in pancreatic expression of TSPO noted here, therefore, could impact on the cholesterol efflux pathway [42] or exert a number of other protective effects sustaining pancreatic function under the challenge of genetic obesity, which could be explored in future studies.

The causal relationships between these proteins and the cholesterol efflux pathway mediated by ABCA1, which can help to sustain β-cell function [19–21,65], were evaluated in BRIN-BD11 insulinoma cells. In interpreting these data, it is important to recognise the distinctions between BRIN-BD11 cells and primary β-cells: electrofusion of rat pancreatic β-cells with immortal RINm5F cells created glucose- and amino acid-responsive clonal insulin-secreting BRIN-BD11 cells [66,67] which are highly proliferative in culture, unlike adult human β-cells [8]. Wild-type BRIN-BD11 cells exhibited glucose-dependent efflux of [^3^H]cholesterol to HDL and human serum, but efflux to apoA-I was not significantly higher than the basal condition, indicating a low capacity for production of nascent HDL via ABCA1.

Overexpression of Cyp27A1 in BRIN-BD11 cells did not affect the biosynthesis, esterification or efflux of cholesterol under basal conditions but enhanced efflux to ApoA-I and human serum in cells previously exposed to native LDL. These findings resonate with our previous study, which suggested that rapidly proliferating BRIN-BD11 cells have suboptimal levels of cholesterol [47], and with that of Fu et al. [24], who demonstrated enhanced induction of LXR-dependent genes involved in the cholesterol efflux pathway in cholesterol-loaded Cyp27-expressing cells, compared with controls. However, ligation of TSPO, using FGIN-1-27, had no impact on the cholesterol efflux pathway in stable populations of Cyp27A1 or EV BRIN-BD11 cells. This finding differs from our studies in macrophages [42] and in retinal pigment epithelium cells [68] which demonstrated that this compound enhances cholesterol efflux to apoA-I, apoE and HDL, an effect which was amplified in cells overexpressing TSPO, and lost after knockdown or genetic ablation of this protein. Equally, the addition of a cAMP analogue to stimulate the expression and activity of StAR, did not stimulate the efflux pathway in either cell line, despite the reported impact of this protein on this pathway, and on lipid metabolism, in macrophages [39–42] and hepatocytes [29,30,46]. This suggests that the mitochondrial cholesterol trafficking complex does not have a major role to play in BRIN-BD11 cells, which do not normally express Cyp27A1; the oxysterols required for ligation of LXRs may therefore derive from the cholesterol biosynthetic pathway rather than from mitochondrial metabolism of excess cholesterol [69]. This may also explain the low capacity for generation of nascent HDL from apoA-I in the absence of a cholesterol ‘load’ observed.

Intriguingly, however, Cyp27A1-expressing BRIN-BD11 cells expressed higher levels of *INS2* than EV controls, even under basal conditions, an outcome consistent with the reported increases in nuclear Pdx-1, and enhanced Pdx-1 binding to the insulin promoter, after treatment of human islets with LXRα agonist, T0901317 [70]. Thus, the expression of *INS2* may be more sensitive to in Cyp27A1-induced changes in oxysterol ‘tone’ than the pathway governing cholesterol removal from these cells. Further, metabolism of oxysterols to more polar products, including bile acids, has been described in macrophages [71]; bile acids are increasingly recognised as signalling molecules with metabolic and endocrine functions [72]. While bile acids have no direct effect on insulin secretion from perfused pancreas [73], it is a limitation of this study that these products were not identified and their physiological impact characterised in the BRIN-BD11 cell lines.

While overexpression of Cyp27A1 alone was sufficient to enhance efflux to apoA-I in macrophages and Chinese Hamster ovary cells [24,74–76], and to reduce hepatic inflammation in LDL receptor^−/−^ mice [77], it is clear that optimal processing of cholesterol and cholesterol metabolites in other cell types, and in BRIN-BD11 cells, requires co-transfection with its redox partners ADX and ADXR [32,59–61,77]. Interestingly, after exposure to native LDL, the level of ADXR declined, relative to β-tubulin, suggesting either a loss of endogenous ADXR expression and/or post-translational degradation of this protein. Despite this, efflux to apoA-I and human serum remained enhanced, reflected in increased expression of ABCA1 protein. Importantly, we did not observe changes in mitochondrial membrane potential or accumulation of mitochondrial cholesterol after overexpression of Cyp27A1/ADXR/ADX. The former finding supports the concept that it is the conversion of cholesterol into oxysterol which is important in modulating the cholesterol efflux pathway in this cell type, rather than the rate of cholesterol delivery to mitochondria, which is known to be modulated by Δψ_m_ in macrophages [78,79] and steroidogenic cells [80]. Thus, in BRIN-BD11 cells, Cyp27A1 appears more effective in stimulating the ABCA1-dependent cholesterol efflux pathway when co-transfected with its redox partners, than when expressed at much higher levels alone, and this effect is also evident following increased flux of LDL cholesterol via the endocytic pathway.

Insulin release into the culture media was increased by the presence of apoA-I, HDL and human serum, with a positive correlation emerging between efflux capacity and insulin release. However, no impact of Cyp27A1/ADXR/ADX overexpression on insulin release, compared with the EV control, could be detected: we speculate that intracellular accumulation of much higher (pathological) levels of cholesterol, or exposure to oxidatively modified forms of LDL, may be required for the full protective effect of this pathway to be manifested. Certainly, insulin granules are the major sites of intracellular cholesterol accumulation in β-cells, and delivery of excess cholesterol to insulin granules can cause defects in their locale, maturation and docking at the plasma membrane [81].

In summary, genetic obesity is associated with hyperinsulinaemia, dyslipidaemia and increased pancreatic expression of Cyp27A1, ADXR, TSPO and LXRα, while overexpression of Cyp27A1 and/or its redox partners promotes the expression of ABCA1 and enhances cholesterol efflux from insulin-secreting cells to apoA-I and HDL. These cellular responses should help to guard against the accumulation of excess intracellular cholesterol which can cause impaired insulin secretion [11–14], particularly since ABCA1 is required for exocytosis of insulin secretory granules [19–21], and β-cell function is sustained by the multiple protective properties of HDL [2–4; 8–10]. The ability to induce the Cyp27A1 pathway may therefore be an important protective mechanism, particularly in ‘pre-diabetic’ individuals characterised by low levels of dysfunctional HDL, in whom insulin resistance requires a compensatory phase of hyperinsulinaemia to maintain normoglycaemia [82]. Once pancreatic β-cells can no longer compensate for insulin resistance, full-blown T2DM with hyperglycaemia develops: it is estimated that one in three adults in England are on this threshold, which may last for up to 10 years [83]. Further work is required to establish the therapeutic potential of the Cyp27A1 pathway in sustaining β-cell capacity during this period.

## Abbreviations

ABC: ATP-binding cassette
ABCA1: ABC transporter A1
ABCG1: ABC transporter G1
ADX: adrenodoxin (ferredoxin)
ADXR: ADX reductase (ferredoxin reductase)
ApoA-I: apolipoprotein A-I
Cyp27A1: cytochrome P450 27 A1/sterol 27-hydroxylase
FGIN-1-27: N,N-Dihexyl-2-(4-flurophenyl)-indole-3-acetamide
HDL: high-density lipoprotein
I.P.: Intra-peritoneal
LXR: liver X receptor
LDL: low-density lipoprotein
pCMV: Plasmid vector utilising the cytomegalovirus promoter to drive gene expression
Pdx1: Pancreatic and duodenal homeobox 1
RPMI: Roswell Park Memorial Institute (cell culture medium)
StAR: steroidogenic acute regulatory protein (StarD1)
T2DM: type 2 diabetes mellitus
TSPO: 18-kDa translocator protein
